# Global status of oral health provision: Identifying the root of the problem

**DOI:** 10.1002/puh2.6

**Published:** 2022-03-29

**Authors:** Kate J. O'Brien, Vivian M. Forde, Michelle A. Mulrooney, Emma C. Purcell, Gerard T. Flaherty

**Affiliations:** ^1^ School of Medicine National University of Ireland Galway Galway Ireland; ^2^ National Institute for Prevention and Cardiovascular Health Galway Ireland

**Keywords:** dental caries, dental tourism, oral health

## Abstract

Dental health is deeply rooted in the primary care of an individual such that an individual's overall health status and behaviours may be determined by their oral health status. Oral diseases are a major cause of morbidity and are among the most prevalent diseases worldwide. Despite the risk these diseases pose to global health, they remain a largely neglected facet of healthcare. In this article, we discuss why predominantly preventable oral diseases persist with high prevalence and suggest how to address the global oral disease epidemic. People living in rural areas are particularly disadvantaged, due to insufficient oral care in remote locations, lack of transport and an overall under‐resourced health care infrastructure. In cases where oral health programmes exist, they are usually directed only at certain groups. Dental tourism has been increasing in popularity over the last two decades. Eastern Europe has become one of the leading destinations for patients seeking dental care across borders. However, negative findings regarding the quality of dental procedures in these destinations have been highlighted in many studies, with some pointing out that only a small fraction of patients received sufficient quality, and that only very simple dental treatments were actually evaluated as cost‐effective. Low‐income countries are hindered not only by lack of dentists, but by lack of public health initiatives such as fluoridation of water and toothpastes. The integration of oral health into medical education and primary care could improve the common risk factor approach between the specialties and improve preventative measures and screening for better patient outcomes and reduction in cost of care.

## INTRODUCTION

Oral diseases are a major cause of morbidity and are among the most prevalent diseases globally. Worldwide, 3.5 billion people are suffering from untreated dental caries, severe periodontitis and edentulism [1]. Dental caries, which are estimated to affect 35% of the world's population, along with periodontal disease, tooth loss and cancers of the lips and oral cavity, are the most prevalent and consequential oral diseases in the modern world [2]. Despite the risk these diseases pose to global health, they remain a largely overlooked and underfunded facet of public healthcare. In this article, we will discuss why predominantly preventable oral diseases persist with such high prevalence and suggest how to put a dent in the global oral disease epidemic.

## GLOBAL BURDEN OF ORAL DISEASE

Dental caries, or tooth decay, are caused by a breakdown of enamel by bacteria on the surface of the tooth. The treatment of caries is based on removing the decay and inserting a filling. It has been shown clinically that caries are preventable, however. If left untreated, dental caries can eventually lead to edentulism. Similarly, periodontal diseases arise due to bacterial action on the teeth and are also attributable to edentulism [1]. Oral diseases in the United States of America (USA) accounted for approximately 1.2 million Disability‐Adjusted Life Years (DALYs) lost in 2019, demonstrating their prevalence and disease burden in society (Table 1) [3].

**TABLE 1:** Age‐standardised percentage of population affected by the main oral disorders for various countries according to the Global Burden of Disease Study 2019 [3]

	Percentage of population affected *(95% Confidence Interval)*			
Country	Caries of deciduous teeth	Caries of permanent teeth	Periodontal diseases	Edentulism
**Brazil**	4.56 *(3.10–6.19)*	22.99 *(19.81–26.46)*	11.97 *(8.69–15.42)*	9.19 *(7.44–11.33)*
**India**	8.40 *(6.76–9.98)*	25.74 *(22.41–29.40)*	16.50 *(12.57–20.16)*	3.14 *(2.40‐ 4.01)*
**Ireland**	8.34 *(6.68–9.91)*	27.65 *(23.51 ‐32.10)*	3.03 *(1.99–4.44)*	6.37 *(5.00–8.13)*
**Malaysia**	7.94 *(6.10–9.71)*	19.96 *(16.79–23.27)*	7.18 *(5.20–9.80)*	5.40 *(4.25–6.89)*
**Nigeria**	6.39 *(4.75–8.59)*	22.13 *(19.00–25.70)*	21.31 *(16.68–25.23)*	1.71 *(1.33–2.20)*
**USA**	7.68 *(6.00–9.46)*	21.72 *(18.75–25.09)*	9.27 *(6.76–12.43)*	4.91 (3.85*–*6.45)

## THE NEED FOR INTERPROFESSIONAL EDUCATION

Dentistry appears to be siloed from mainstream medical practice, despite the obvious overlap between the two fields. The integration of oral health into undergraduate medical education and primary care could improve the common risk factor approach between the specialties and improve preventative measures and screening for better patient outcomes and reduction in cost of care. Likewise, dentists should be well grounded in general medicine and be encouraged to take a more prominent role in preventive medicine, for example by educating patients with periodontitis about the need to screen for other risk factors for cardiovascular disease. Many universities do not have both medical and dental schools and there is a need to develop closer interprofessional links between these healthcare pillars. A curriculum in interprofessional oral health care would be a useful starting point in order to achieve greater integration between dentistry and medicine.

## COSTS OF ORAL DISEASE

One study estimated in 2010 that direct treatment costs due to dental diseases accounted for 4.6% of the global health expenditure, amounting to US$298 billion. However, this study highlighted the absence of comprehensive information about dental expenditure worldwide. This lack of reporting and collection of data surrounding oral health negatively impacts efforts to estimate the economic burden of oral diseases and the benefits associated with their treatment. In addition to these direct treatment costs, there are significant indirect costs of untreated oral conditions to be considered. It has been estimated that oral diseases account for productivity losses of greater than $1 billion annually in Canada alone. This loss of productivity globally is due mainly to absenteeism from school and work [4].

## ORAL HEALTH AS A PUBLIC HEALTH PRIORITY

A 2020 study showed that, on average, 7.3% of the annual gross domestic product (GDP) of low‐, middle‐ and high‐income countries was allocated to healthcare and just 1.3% of this was allotted towards oral health, highlighting the neglect and lack of priority for this area. Each country must ensure that no one should suffer from financial austerity in an attempt to receive the quality health care they need according to the World Health Organisation (WHO) policy on Universal Health Coverage. Despite this policy, government involvement was shown to be weak in low‐income countries, with 30% of all countries reporting that people received support from private health insurance [5]. The USA is considered a high‐income country, yet approximately 75 million of its citizens are enrolled in the country's low‐income insurance program Medicaid. One such citizen was 12‐year‐old Deamonte Driver, who died in Maryland in 2007 as a result of an untreated oral infection that led to fatal meningitis. His family was left with a hefty and avoidable hospital bill. Medicaid users, like Deamonte's family, face significant obstacles in accessing oral health care [6].

## BARRIERS TO ACCESSING ORAL HEALTH CARE

Many of these barriers prevent people from accessing oral healthcare worldwide. Lack of affordability and the significant costs of oral treatment leave many unable to afford basic oral care. Due to privatisation, the proportion of the population unable to access private dental care is increasing. Lack of adequate universal insurance coverage for oral disease prevention and treatment presents a significant challenge to the provision of high quality oral healthcare globally. Those living in rural areas are particularly disadvantaged, due to insufficient oral care in remote locations, lack of transport and an overall under‐resourced health care infrastructure. In cases where oral health programmes exist, they are usually directed only at certain groups. An example of this is school dental visit programmes targeting school‐age children. However, these programmes have now been discontinued in many places, including most Eastern European countries [7]. Vulnerable adults and other minorities are often forgotten about, with many facing racial prejudice or perceived inferior care. Among those who may be able to afford and access dental care, there are various personal factors that may prevent them from availing of care, including anxiety or fear, and lack of interest and time. Lack of perceived need means many are unwilling to pay for regular check‐ups if they do not think there is anything wrong with their teeth [8].

## INTERNATIONAL DENTAL TOURISM

From these barriers to oral care, the relatively new phenomenon of dental tourism has emerged. Dental tourism has been increasing in popularity over the last two decades. Eastern Europe has become one of the leading destinations for patients seeking dental care across borders. Some promotional websites advertise that top quality dental procedures in Hungary may cost about 50% of the same procedures in Western European countries. Dentists practising in Hungary identified monetary factors as the main incentive for foreign patients. However, concerning findings regarding the quality of dental procedures in these destinations have been highlighted in many studies, with some pointing out that only a small fraction of patients received sufficient quality dental work, and that only very simple dental treatments were actually evaluated as cost‐effective. Patient safety concerns have also been widely discussed in the dental tourism debate, with barotrauma risks and lack of follow‐up care being of utmost concern [9]. With such vast differences seen across European Union (EU) member countries alone in their accessibility to, and quality of dental care, one has to consider the bigger picture of inequalities seen across the globe.

## GLOBAL INEQUALITIES IN ORAL HEALTH CARE

There exist major inequalities between high‐ and low‐income countries with regards to the provision of primary dental care and emergency oral care. Low‐income countries (LIC) have substantially fewer registered dentists than both middle‐ (MIC) and high‐income countries (HIC), with the population per dentist in LIC being 152,721:1 compared to 1708:1 in HIC. In the LIC, half of the dentists work in public health care compared to just one‐fifth of dentists in both MIC and HIC. Pit‐and‐fissure sealants on the permanent teeth of schoolchildren were less frequently used in LIC when compared to the HIC. Pit‐and‐fissure sealants are used to provide a physical barrier, thereby inhibiting microorganism and food particle accumulation and thus either preventing caries initiation or progression. The gap between HIC and LICs when it comes to access to dentists and dental health care, both primary and emergency, is wide.

Low‐income countries are hindered not only by lack of dentists, but by lack of public health initiatives, too. These include fluoridation of water and availability of toothpastes. In LIC, no one is exposed to adequate amounts of fluoride in the water and only 30.8% benefit from fluoride fortified toothpaste, in comparison to 20.9% of the population accessing fluoridated water and 81.5% accessing fluoridated toothpaste in the HIC. The lack of access to fluoridated water in low‐income countries is a major risk factor for the development of dental diseases and it stems from the lack of piped drinking water and the lack of funding for the installation of these programmes [5].

## APPROACHES TO ORAL DISEASE PREVENTION

Many other shared risk factors, which include free sugar and alcohol consumption and tobacco use, are common to both oral diseases and many other non‐communicable diseases. Free sugar consumption is of particular concern due to its contribution to the prevalence of caries, obesity and associated conditions such as type 2 diabetes. Revised public health policies and tighter restrictive legislation surrounding sugar, alcohol and tobacco are necessary to decrease the prevalence of oral diseases around the world [2]. Greater efforts to improve availability and uptake of human papillomavirus (HPV) vaccination globally could also help to decrease the incidence of oropharyngeal cancer related to HPV infection [7]. The recently published World Health Organization draft global strategy on tackling oral diseases provides a framework for monitoring progress towards measurable goals [10]. It sets out six guiding principles, namely developing a public health approach to oral health; integrating oral health in primary heath care; reforming the oral health workforce to respond to population needs; creating a culture of person‐centred oral health care with a focus on oral health literacy, shared decision‐making and self‐management; providing age‐appropriate oral health across the life course; and harnessing digital technologies for oral health, including the development of telehealth.

## SYSTEMIC COMPLICATIONS OF NEGLECTED ORAL HEALTH

Dental health is deeply rooted in the primary care of an individual. An individual's overall health status and behaviours may be determined by their oral health status. A 2014 study proved that there appeared to be a link between the presence of heart disease, diabetes, or cancer and both oral health conditions and death. The study also highlighted the link between having more than one oral health condition and a high likelihood of mortality [11]. If dental caries are not given appropriate treatment, they may result in various oral complications such as periodontitis and tooth loss, but may also have a systemic effect resulting in bacterial and bloodstream infections such as infective endocarditis, sepsis, and possibly even death.

## CONCLUSION

It is evident that a truly holistic approach cannot be taken to healthcare without an increased consideration for oral health. While obvious inequalities exist in the accessibility of oral care in low‐income countries compared to high‐income countries, oral diseases persist with high prevalence among all populations, highlighting the global neglect of oral health. As oral diseases impose immense economic burden on individuals affected and on labour markets in terms of productivity loss, improvements in population oral health would confer substantial economic benefits. We propose that these improvements in population oral health can be attained by public oral health policy reform, with special emphasis placed on prevention of oral diseases, and by the integration of oral care with primary care provision.

## CONFLICT OF INTEREST

None declared.

## ETHICS APPROVAL

As this is a perspective article, no ethics approval was required.

## AUTHOR CONTRIBUTIONS

GTF was responsible for study conception. Kate J. O'Brien, Vivian M. Forde, Michelle A. Mulrooney and Emma C. Purcell contributed equally to the preparation of the first draft of the manuscript, which was edited for significant intellectual content by Gerard T. Flaherty. All authors read and approved the final version of the manuscript.

## Data Availability

No database or primary data were used in preparing this manuscript.

## References

[1] Watt RG , Daly B , Allison P , et al. Ending the neglect of global oral health: time for radical action. Lancet. 2019;394(10194):261‐272. 31327370 10.1016/S0140-6736(19)31133-X

[2] Peres MA , Macpherson LMD , Weyant RJ , et al. Oral diseases: a global public health challenge. Lancet. 2019;394(10194):249‐260. 31327369 10.1016/S0140-6736(19)31146-8

[3] Institute of Health Metrics and Evaluation . 2019. GBD Compare Viz Hub. Available at: https://vizhub.healthdata.org/gbd‐compare/. (Accessed November 10, 2021).

[4] Listl S , Galloway J , Mossey PA , Marcenes W . Global economic impact of dental diseases. J Dent Resh. 2015;94(10):1355‐1361. 10.1177/0022034515602879 26318590

[5] Petersen PE , Baez RJ , Ogawa H . Global application of oral disease prevention and health promotion as measured 10 years after the 2007 World Health Assembly statement on oral health. Community Dent Oral Epidemiol. 2020;48(4):338‐348. 32383537 10.1111/cdoe.12538 PMC7496398

[6] Mofidi M , Rozier RG , King RS . Problems with access to dental care for medicaid‐insured children: what caregivers think. Am J Public Health. 2002;92(1):53‐58. 11772761 10.2105/ajph.92.1.53 PMC1447388

[7] Northridge ME , Kumar A , Kaur R . Disparities in access to oral health care. Annu Rev Public Health. 2020;41:513‐35. 31900100 10.1146/annurev-publhealth-040119-094318 PMC7125002

[8] Krishnan L , Aarthy CS , Kumar PDM . Barriers to access dental care services among adult population: a systematic review. J Global Oral Health. 2020;3:54‐62.

[9] Österle A , Balázs P , Delgado J . Travelling for teeth: characteristics and perspectives of dental care tourism in Hungary. Br Dent J. 2009;206(8):425‐428. 19396208 10.1038/sj.bdj.2009.308

[10] World Health Organization . 2021. WHO discussion paper: draft global strategy on oral health. Available at: https://www.who.int/publications/m/item/who‐discussion‐paper‐draft‐global‐strategy‐on‐oral‐health (Accessed March 7, 2022).

[11] Kim JKi , Baker LA , Davarian S , Crimmins E . Oral health problems and mortality. J Dent Sci. 2013;8(2):115‐120. 10.1016/j.jds.2012.12.011. PMC3885153 24416472

